# Demeclocycline Reduces the Growth of Human Brain Tumor-Initiating Cells: Direct Activity and Through Monocytes

**DOI:** 10.3389/fimmu.2020.00272

**Published:** 2020-02-21

**Authors:** Susobhan Sarkar, Yibo Li, Reza Mirzaei, Khalil S. Rawji, Candice C. Poon, Jianxiong Wang, Mehul Kumar, Pinaki Bose, V. Wee Yong

**Affiliations:** ^1^Department of Clinical Neurosciences, The Hotchkiss Brain Institute and the Arnie Charbonneau Cancer Institute, University of Calgary, Calgary, AB, Canada; ^2^Department of Oncology, The Hotchkiss Brain Institute and the Arnie Charbonneau Cancer Institute, University of Calgary, Calgary, AB, Canada; ^3^Department of Biochemistry and Molecular Biology, The Hotchkiss Brain Institute and the Arnie Charbonneau Cancer Institute, University of Calgary, Calgary, AB, Canada; ^4^Department of Surgery, The Hotchkiss Brain Institute and the Arnie Charbonneau Cancer Institute, University of Calgary, Calgary, AB, Canada

**Keywords:** glioma, innate immunity, monocytes, macrophages, microglia, stem cells

## Abstract

Myeloid cells that infiltrate into brain tumors are deactivated or exploited by the tumor cells. We previously demonstrated that compromised microglia, monocytes, and macrophages in malignant gliomas could be reactivated by amphotericin-B to contain the growth of brain tumorinitiating cells (BTICs). We identified meclocycline as another activator of microglia, so we sought to test whether its better-tolerated derivative, demeclocycline, also stimulates monocytes to restrict BTIC growth. Monocytes were selected for study as they would be exposed to demeclocycline in the circulation prior to entry into brain tumors to become macrophages. We found that demeclocycline increased the activity of monocytes in culture, as determined by tumor necrosis factor-α production and chemotactic capacity. The conditioned medium of demeclocycline-stimulated monocytes attenuated the growth of BTICs generated from human glioblastoma resections, as evaluated using neurosphere and alamarBlue assays, and cell counts. Demeclocycline also had direct effects in reducing BTIC growth. A global gene expression screen identified several genes, such as DNA damage inducible transcript 4, frizzled class receptor 5 and reactive oxygen species modulator 1, as potential regulators of demeclocycline-mediated BTIC growth reduction. Amongst several tetracycline derivatives, only demeclocycline directly reduced BTIC growth. In summary, we have identified demeclocycline as a novel inhibitor of the growth of BTICs, through direct effect and through indirect stimulation of monocytes. Demeclocycline is a candidate to reactivate compromised immune cells to improve the prognosis of patients with gliomas.

## Introduction

Malignant gliomas are brain tumors that arise from within the central nervous system (CNS). The most aggressive form, glioblastoma multiforme, has a dismal prognosis with a median survival of 15 months; <10% of patients survive beyond 5 years (1–3). The poor prognosis of malignant gliomas is attributed in part to the existence of glioma stem cells, also called brain tumor-initiating cells (BTICs) (4–9). BTICs are resistant to therapies as they continue to seed and form new tumor foci in the brain. BTICs have been shown to contribute to the tumourigenesis and recurrence of gliomas (10), particularly due to their high chemo- and radio-resistance (11, 12).

Surrounding BTICs *in situ* are microglia, which are innate immune cells of the CNS and macrophages that have infiltrated as monocytes from the circulation (13–16). These cells are thought to be initially recruited to eradicate the tumor by stimulating apoptosis of glioma cells (17) and by secreting inflammatory factors that prevent glioma growth and invasiveness (18). However, glioma cells have been shown to induce an immunosuppressive phenotype that in turn enhances tumor growth. For example, glioma cells have been shown to secrete periostin, which selectively recruits macrophages with an immunosuppressive profile (19). Furthermore, interactions between glioma and macrophages/microglia can lead to promotion of tumor growth (20–22). These immune cells have been shown to enhance tumor CCL21 expression, which facilitates tumor immune escape (23). Notably, BTICs also interact with macrophages and microglia within the tumor microenvironment, inducing an immunosuppressive macrophage/microglia cell profile that leads to promotion of tumor invasion (24, 25). We made the discovery that microglia, monocytes, and macrophages derived from glioma patients are deficient in their capacity to reduce the growth of BTICs (26).

Based on the above discussion, activating or reprogramming immune cells may represent an approach to curb BTIC growth (27–29). Kees et al. (30) demonstrated that stimulation of microglia with toll-like receptor-3 agonist, poly(I:C), prior to co-culture with tumor cells promotes microglia tumouricidal activity *in vitro*. However, direct poly (I:C) treatment was ineffective in glioma patients (30).

A recent study has shown that manipulation of RNA regulator in tumor-associated microglia and macrophages stimulates anti-tumor immunity and reduces glioma growth (31). More recently, we found that the compromised monocyte, macrophages and microglia from patients with glioma could be reactivated by amphotericin B to reduce BTIC growth in culture and to prolong the lifespan of mice with intracranial patient-derived BTIC xenografts (26). Despite these promising results, amphotericin B may not find clinical utility in gliomas as it has significant acute and chronic toxic side effects ranging from hypoxia to nephrotoxicity (32).

We discovered the pro-activation capacity of amphotericin B on microglia during a screen of a 1,040-drug library (33). During that screen, we discovered another stimulator of the activity of microglia in culture, as measured by TNF-α production: meclocycline, a tetracycline antibiotic. Meclocycline has significant toxicity and is limited to topical use, but a derivative, demeclocycline, can be administered systemically (34) (www.drugs.com). Demeclocycline is used clinically as a prescription medication to treat susceptible bacterial infections, as well as off-label to manage chronic syndrome of inappropriate secretion of anti-diuretic hormone (SIADH).

Here, we have evaluated the effects of demeclocycline on BTIC growth either through direct mechanisms or indirectly through the stimulation of monocytes. We chose monocytes for study as systemic monocytes would be exposed to demeclocycline prior to their infiltration into brain tumors as macrophages to influence BTIC properties. Our results suggest the potential utility of demeclocycline in glioblastoma.

## Materials and Methods

### Isolation and Culture of Monocytes and Macrophages

Human monocytes were isolated from the venous blood of adult healthy individuals as described elsewhere (26). Briefly, monocytes (100,000 cells/well/100 μl) following isolation were plated in RPMI medium supplemented with 20% human serum in 96 well plates. After 24 h, cells were switched to BTIC medium. Cells were transferred to BTIC medium an hour prior to treatment. Treatment involved administering each drug at different concentrations with or without LPS (100 ng/ml). Briefly, monocytes were treated with demeclocycline (10 or 1 μM) for 48 h in BTIC medium and conditioned media were collected. Bone marrow-derived macrophages (BMDM) were isolated from mice as described elsewhere (35). Unless otherwise stated, BMDM cells were plated at 30,000 cells in AIMV medium for collection of conditioned media (see below), or for assessment of their activity.

### Evaluation of Activity of Monocytes and Macrophages in Response to Demeclocycline Treatment

We utilized the level of tumor necrosis factor-α (TNF-α) as a first indicator of cellular activity. Following 24 h treatment with demeclocycline (10 μM, Sigma) with or without IFNγ (100 ng/ml)/IL-1β (100 ng/ml) (Peprotech) or LPS (100 ng/ml), the conditioned medium was collected for TNF-α ELISA following manufacturer's instructions (Life Technologies Invitrogen).

#### Chemotaxis Assay

Human monocytes were treated with demeclocycline (10 μM). After 1 h of incubation at 37°C with 5% CO_2_, IFNγ (100 ng/ml)/IL-1β (100 ng/ml) was added. After 24 h, human monocytes were harvested and resuspended in RPMI 1640 media supplemented with 2% penicillin/streptomycin, 10% fetal bovine serum, L-glutamine, and 1 mM sodium pyruvate. Two hundred thousand cells were plated onto the filters of 5 μm pore size ChemoTx plates (NeuroProbe). Recombinant human CCL2 (Peprotech) (10 ng/ml) was diluted in supplemented RPMI 1640 media and 300 μl/well was added into wells below the filter so as to provide a chemotactic stimulus. Two controls were used in this assay. The first control was medium only. The second one was chemokinetic control where the cells plated onto the filter contained the 10 ng/ml of CCL2 as in the underlying well. To obtain a standard curve, halving numbers of cells were plated ranging from 0 to 200,000. Cells were incubated at 37°C in humidified air with 5% CO_2_ for 16 h. They were then washed off the top of the filter and the plate spun at 1,400 rpm for 10 min at room temperature. One hundred and fifty microliter of the media was discarded in the microplate and replaced with 15 μl of alamarBlue® (Invitrogen). The plate was then placed at 37°C in humidified air with 5% CO_2_ for 4 h and signal was read at 570 nm. This assay was also conducted with mouse BMDM.

#### Human Neuron Toxicity Assay

Brain tissue from fetuses legally aborted at 15–20 weeks was used to isolate human neurons. The use of the brain cells was conducted with ethics approval from the University of Calgary human ethics committee. The neurons were isolated by removal of the meninges followed by mechanical dissociation of the tissue. Tissue was then digested in DNase (6–8 mL of 1mg/mL; Roche), 4 mL 2.5% trypsin and 40 mL PBS (37°C, 25 min). Digestion was quenched by the addition of 4 mL of fetal bovine serum (FBS) after which the solution was filtered through a 132 μm filter. The solution was then centrifuged three times at 1,200 rpm for 10 minutes. Cells were then cultured in medium supplemented with 10% FBS, 1 μM sodium pyruvate, 10 μM glutamine, 1x non-essential amino acids, 0.1% dextrose, and 1% penicillin/streptomycin (Invitrogen). Cells were plated in poly-L-ornithine-coated T75 flasks and cultured for two cycles in medium consisting of 25 μM cytosine arabinoside (Sigma-Aldrich). The inclusion of cytosine arabinoside inhibits astrocyte proliferation. To complete experiments, cultures enriched (~80%) for neurons were re-trypsinized and plated in poly-L-ornithine coated 96-well plates at a density of 100,000 cells/well. After 24 h, medium was changed to serum-free AIM V medium. After another 24 h, demeclocycline (10 μM) was added to the neurons. Cells were fixed 24 h after with 4% paraformaldehyde and stained for MAP-2 (mouse anti-MAP-2 antibody; clone HM-2; Sigma; M4403; 1:1,000) and Hoeschst S769121. Cells were imaged with an ImageXpress® imaging system (Molecular Devices) and quantified using MetaXpress® (Molecular Devices).

### Culture of Human BTICs

BTICs were isolated from resected specimens of patients with glioblastoma (7, 9, 26, 36). We used three BTIC lines derived from glioma patients designated BT012, BT025, and BT048. These lines had different genetic mutations (26) including BT012: EGFR wildtype (wt), p53 wt, PTEN mutant (mt, frameshift in codon 328); BT025: EGFR wt, p53 mt (T125R), PTEN mt (G129R); and BT048: EGFR mt (K294R, G598V), p53 wt, PTEN wt. BT025 and BT048 were employed in the majority of experiments since they were characterized extensively in our previous study (6–9, 26). To propagate the lines, BTICs were dissociated and plated into T25 flasks at regular intervals and grown in serum free culture medium supplemented with epidermal growth factor and fibroblast growth factor-2 in 5% CO_2_ as described elsewhere (36, 37); we refer to this as BTIC medium. The lines with higher passage numbers were checked for stemness and self-renewal property (data not shown). All experiments with human cells or resected brain specimens were conducted with approval from the Conjoint Health Research Ethics Board, University of Calgary, with informed consent from the human subjects.

We documented passage number (denoted by P) after thawing a new vial of cells from liquid nitrogen and kept a record of the number of subsequent passaging. Frozen stocks of BTIC cells were made as soon as possible from previously thawed BTIC lines, to avoid cell changes, contamination, etc. for the next set of experiments. Every time a vial (of BTIC line) was thawed, new stocks were made within a week or two of the growing culture. Thus, the BTICs were usually frozen between P2-P3 of a newly thawed culture. We used BTICs (after thawing) from the expanding and/or growing cultures for experiments between P2 and P10. The lines with higher passage numbers (P8–P10) were checked for stemness using stemness markers such as nestin, SOX-2 and Musashi-1 by FACS analysis, and by the ability of dissociated single cells to form spheres. Importantly, BTICs were also sent for sequencing at regular intervals to verify the identity of lines (to ascertain their genetic background with the parental line).

### Evaluation of BTIC Growth

For neurosphere assay, BTIC cells (10,000 cells/well/100μl of serum free BTIC medium) were plated into 96-well plates (7, 26, 36). The resultant number of spheres above the 60 μm diameter cutoff, a convenient parameter to describe growth characteristics, was monitored after 72 h by photographing multiple fields per well with subsequent image analyses. Where total cell number was documented, the medium containing the BTIC spheres was collected, centrifuged, resuspended in 25 μL of Accumax™, mixed with Trypan Blue (1:1) and counted using a TC20™ automated cell counter (Bio-Rad).

An alamarBlue® assay was also used to evaluate growth. At predetermined times, alamarBlue® dye (1:10, Life Technologies) was added to each well of cells for 4–6 h after which readings were taken with a Spectra Max Gemini XS (emission wavelength = 590 nm, excitation wavelength = 544 nm; Molecular Devices). Finally, annexin V–propidium iodide staining was carried out and analyzed using FACS as described before (26).

### Drugs Used

Demeclocycline hydrochloride, tetracycline hydrochloride, and oxytetracycline hydrochloride stock solutions were prepared (10 mM in DMSO; all chemicals from SigmaAldrich) and diluted immediately prior to treatment of cells. While, demeclocycline was used at 1, 5, and 10 μM final concentrations, tetracycline and oxytetracycline were used at 10 μM only. All dilutions from stock were done in BTIC medium.

### Microarray and Bioinformatics

BTICs (BT012, BT025, and BT048) were treated with 10 μM demeclocycline for 6 h. RNA was then extracted using a mirVana miRNA Isolation Kit (Ambion, Austin, USA) according to the manufacturer's protocol. Total RNA was purified with RNeasy Plus Micro Kit (Qiagen, Valencia, USA) to remove genomic DNA. The RNA quality of integrity number (RIN) was measured with Agilent RNA Nano Chips on 2,100 Bioanalyzer (Agilent Technologies, Santa Clara, USA). The total of 250 ng of RNA for each sample with RIN higher than 9 was labeled with WT Express Kit (Ambion) and hybridized to Affymetrix GeneChip Human Gene 2.0 ST Array at 45°C for 16 h. Arrays were stained and washed on Affymetrix GeneChip Fluidics_450 following manufacturer's protocol and scanned with Affymetrix GeneChip Scanner 3,000 7G System.

For data analysis, array data files were generated with GeneChip® Command Console® Software (AGCC) and statistical analyses were carried out in GeneSpring^TM^ (Agilent Technologies). The fold change between treatment and control was based on the *p* < 0.05 from Ttest of unpaired samples.

### Statistical Analyses

The one-way ANOVA with *post-hoc* Tukey's comparisons was used for multiple group comparisons unless otherwise mentioned, while the *t*-test was used for comparisons of two groups. We used GraphPad Prism software for statistical analysis.

## Results

### Demeclocycline Promotes the Activity of Monocytes and Macrophages and Is Not Toxic to Neurons

**Figure 1:**
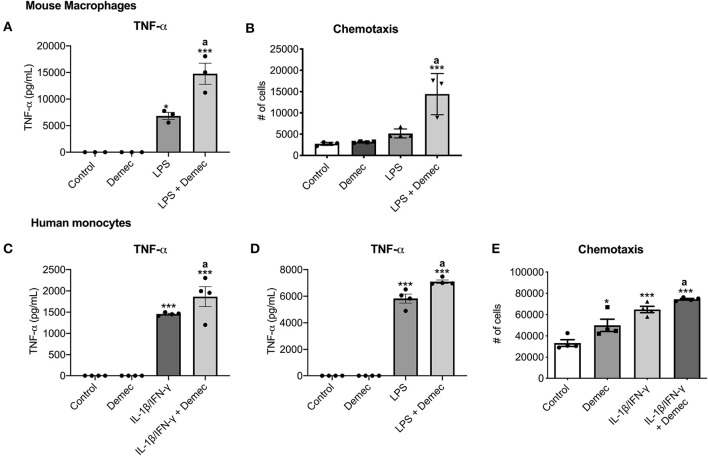
Demeclocycline enhances TNF-α production and modulates monocyte functions. **(A,B)** For mouse BMDM, demeclocycline increases TNF-α level and CCL2-directed chemotaxis in LPS-stimulated condition. **(C,D)** ELISA for TNF-α in human cells shows a further elevation of cytokine in IL-1β/IFN-γ **(C)** or LPS **(D)** stimulated monocytes by demeclocycline. **(E)** The chemotaxis of human monocytes toward a CCL2 gradient is promoted by demeclocycline in IL-1β/IFN-γ-stimulated condition. In all cases, demeclocycline was used at 10 μM. *p < 0.05, ***p < 0.001 compared to control; ^a^significantly different from IL-1β/IFN-γ or LPS in their respective panels (1-way ANOVA with Tukey's multiple comparisons test). Error bars represent s.e.m.

While we identified meclocycline as an activator of microglia (33), it was necessary to confirm that its better tolerated derivative, demeclocycline, also has such activity. We first carried out some preliminary studies with mouse bone marrow derived macrophages (BMDM). Notably, demeclocycline alone did not induce mouse macrophages to increase TNF-α production; however, when combined with LPS, demeclocycline further promoted the production of TNF-α (Figure 1A). Moreover, as another index of activity, demeclocycline promoted the chemotaxis (Figure 1B) of stimulated macrophages when combined with LPS.

To corroborate the above findings of mouse cells, we evaluated whether human cells were responsive to demeclocycline. We investigated monocytes isolated from healthy human donors, as these cells would be exposed to demeclocycline in the circulation after systemic administration and could then traffic into the glioma microenvironment as macrophages. We found that human monocytes under basal conditions did not elevate their production of TNF-α, an index of activity, in the presence of demeclocycline alone. However, when human monocytes were exposed to IL1β/IFN-γ (Figure 1C), cytokines that are elevated in glioma subjects (38), or to the toll-like receptor-4 ligand LPS (Figure 1D), demeclocycline elicited a further increase in TNF-α levels in activated cells.

The migration of monocytes to a chemokine source, chemotaxis, constitutes another index of cellular activity. In IL-1β/IFN-γ-primed conditions, we noted that demeclocycline promoted the chemotaxis of human monocytes to CCL2 (Figure 1E); alone, demeclocycline had some enhancing activity.

### Demeclocycline-Treated Monocytes Reduce BTIC Growth

**Figure 2:**
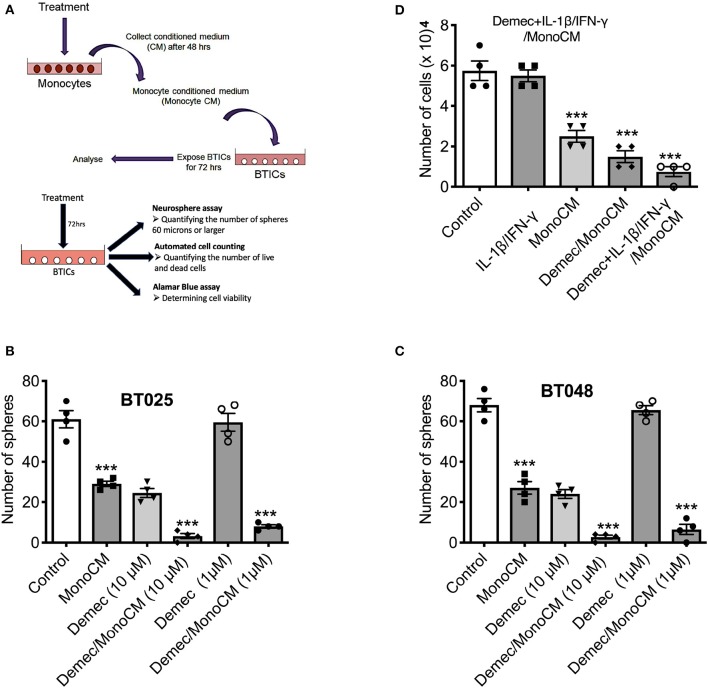
Demeclocycline activated monocytes reduces BTIC growth in culture. **(A)** Generation of monocyte-conditioned media. **(B,C)** Untreated human monocytes (MonoCM) or monocytes exposed to demeclocycline (Demec/MonoCM) attenuate BTIC growth compared to basal control after 72 h as measured through neurosphere assay. Although demeclocycline alone reduced the BTIC growth at 10 μM, it was ineffective at 1 μM in both lines. Notably, Demec/MonoCM had robust effect on BT025 and BT048 cells, and was significantly greater than that of either MonoCM or demeclocycline alone. **(D)** Demec/MonoCM also reduced the total cell counts in BT048 line, and this BTIC growth inhibitory effect was promoted in IL-1β/IFN-γ treated monocyte cultures. ***p < 0.001 compared to control (1-way ANOVA with Tukey's multiple comparisons); *n* = 4 for all groups. Error bars represent s.e.m.

Our previous study (26) found that the capacity of microglia, monocytes and macrophages to reduce BTIC growth could be elicited through the conditioned medium of these cells. Thus, monocytes were isolated from the peripheral venous blood of healthy volunteers and conditioned media were generated (Figure 2A). To determine the capacity of demeclocycline as a novel stimulator of innate immunity to reduce BTIC growth, BTIC lines plated at 10,000 cells per well in 96-well plates were exposed to conditioned medium from untreated monocytes (MonoCM) or to the conditioned medium of monocytes exposed for 48 h to 10 μM demeclocycline (Demec/MonoCM). Reproducing previous results (26), MonoCM reduced the growth of the BT025 and BT048 lines (Figures 2B,C) in sphere-forming assays; importantly, Demec/MonoCM decreased the growth of BTICs further (Figures 2B,C) and there was an additional effect on reducing BTIC growth when conditioned medium from monocytes exposed to both demeclocycline and IL-1β/IFN-γ (Demec + IL-1β/IFN-γ/MonoCM) was used (Figure 2D).

In these experiments, we noted that demeclocycline (10 μM) added directly to BTICs in the absence of monocyte intermediary was sufficient to reduce BTIC growth, suggesting that the medication may affect BTICs in 2 ways: through monocyte intermediary and directly on BTICs.

### Demeclocycline Directly Affects the Growth of BTICs

**Figure 3:**
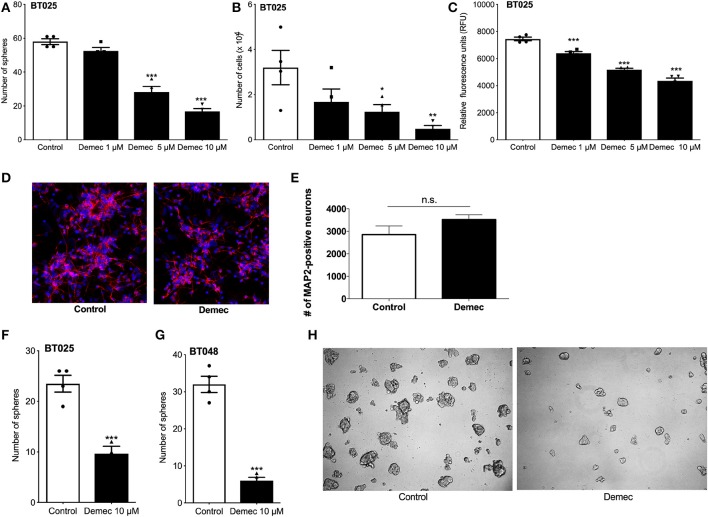
Demeclocycline has a direct impact on BTIC growth reduction. **(A–C)** Treatment of BT025 with varying concentrations of demeclocycline reduced BTIC growth in culture as evident by reduced sphere formation **(A)**, total number of cells **(B)**, and alamarBlue® assay **(C)**. Results are reproduced with BT048 (data not shown). Error bars represent s.e.m. *p < 0.05, **p < 0.01, ***p < 0.001 compared to controls (1-way ANOVA with Tukey's multiple comparisons test). **(D,E)** Representative images of MAP2-positive neurons untreated or treated with demeclocycline, and quantification of MAP2-positive neurons demonstrates that demeclocycline was not toxic to human neurons. **(F,G)** Demeclocycline reduced the number of spheres above the 60 μm cutoff when applied to growing spheres 3 days after their formation from singly dissociated cells. Error bars represent s.e.m (*n* of 4). ***p < 0.001 (two-tailed Student's *t*-test). (**H)** Representative images showing reduced sphere size in demeclocycline-treated BTICs (BT048) compared to control at 5 days.

Because the above results suggest that demeclocycline alone reduced BTIC growth, we sought to investigate its direct role further. We subjected BTICs to different concentrations of demeclocycline and found that 5 and 10 μM decreased sphere formation and cell number (Figures 3A,B); an effect on BTIC could be documented for 1 μM demeclocycline using the alamarBlue® assay (Figure 3C). Notably, demeclocycline at 10 μM concentration had selective efficacy on BTICs as it was without obvious toxicity to non-transformed CNS cells such as microtubule associated protein-2 (MAP-2) labeled neurons (Figures 3D,E).

As the above experiments involved the treatment of freshly dissociated BTIC lines with demeclocycline to determine whether the medication reduced sphere formation and other indices of BTIC growth, we next addressed whether demeclocycline affected BTIC spheres that were already well-formed. We found that when demeclocycline (10 μM) was added to growing spheres 3 days after their formation from singly dissociated cells, the drug still reduced the further growth of spheres of the BT025 and BT048 lines (Figures 3F–H).

Overall, our results suggest that demeclocycline can control BTIC growth in two ways: using monocytes as an intermediary, and directly by affecting the proliferation and sphere-forming capacity of BTICs.

### Mechanisms of Demeclocycline-Mediated BTIC Growth Reduction

**Figure 4:**
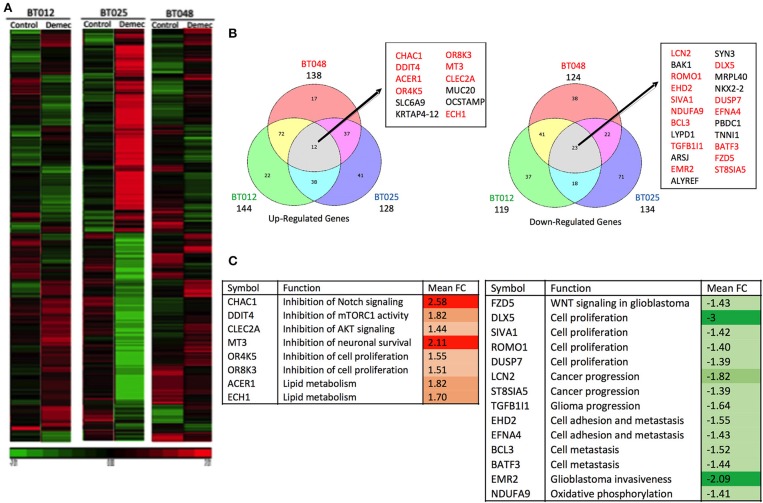
Microarray analyses of BTICs exposed to demeclocycline. **(A)** Heat map depicting patterns of changing gene expression in three genetically divergent glioblastoma patient-derived BTIC lines after 6 h of demeclocycline treatment, compared to respective controls; red represents elevation while green displays genes that are reduced by demeclocycline (GEO accession number GSE81515). **(B)** Venn diagrams comparing up-regulated (fold change, FC ≥ 1.3) and downregulated (FC≤ −1.3) genes in the three BTIC lines following treatment with demeclocycline. Genes intersecting in all 3 sets are noted in box; genes which may be involved in tumor progression are represented in red. Diagrams are generated by Bioinformatics.lu software. **(C)** Genes with possible role in tumor progression are shown. Up-regulated genes (relative to untreated cells) are represented in red; down-regulated genes (relative to untreated cells) are represented in green. Mean fold change (FC) is calculated for the three BTIC lines following treatment with demeclocycline vs. no treatment. The intensity of the colors represents the strength of the gene deregulation.

**Figure 5:**
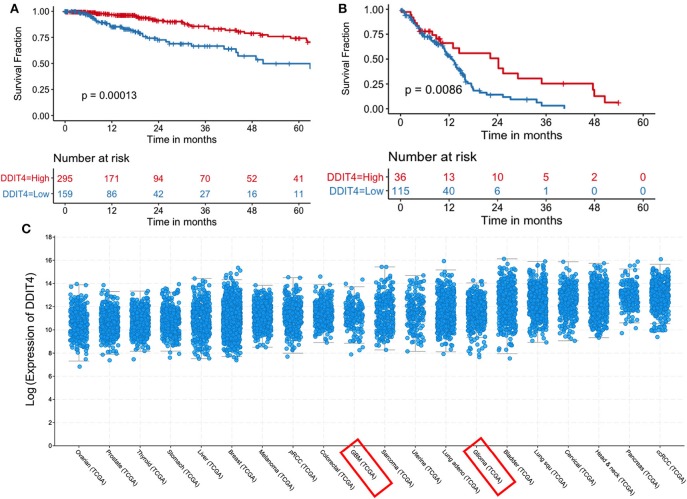
Increased DDIT4 expression is associated with improved survival in gliomas. **(A,B)** Kaplan–Meier curves showing the association between *DDIT4* mRNA expression and overall survival in TCGA **(A)** low-grade glioma and **(B)** glioblastoma patients. **(C)** *DDIT4* mRNA expression was plotted across 20 major solid tumor types in TCGA database arranged by increasing median expression (pRCC, Papillary Renal Cell Carcinoma; ccRCC, clear cell renal cell carcinoma; Lung squ, lung squamous cell carcinoma; GBM, glioblastoma).

We sought to obtain insights into the mechanisms by which demeclocycline directly reduces BTIC growth. We subjected 3 BTIC lines to microarray analyses and identified 301 genes (with a cutoff fold change = 1.3) amongst the three lines that were commonly affected by demeclocycline treatment compared to controls (Figure 4A and Supplementary Table 1) (GEO accession number GSE81515). Analysis of the array data using gene-ontology criteria with Panther Classification System bioinformatics software identified several genes that were up or down regulated with demeclocycline treatment (Figure 4B and Supplementary Figure 1). Notably, we found a number of genes known to be involved in glioma progression, invasiveness, signaling or cancer progression that were down regulated by demeclocycline. These include transforming growth factor β1 induced transcript 1 protein (TGFB1I1) (39), frizzled class receptor 5 (FZD5) (40), epidermal growth factor module-containing mucin-like receptor 2 (EMR2) (41), reactive oxygen species modulator 1 (ROMO1) (42) and B cell lymphoma 3 protein (BCL3) (43) (Figure 4C). In contrast, genes that negatively regulate notch signaling, mTOR activity or AKT such as ChaC Glutathione Specific Gamma-Glutamylcyclotransferase 1 (CHAC1), DNA Damage Inducible Transcript 4 (DDIT4) and C-Type Lectin Domain Family 2 Member A (CLEC2A) were up regulated. With demeclocycline treatment in three BTIC lines (Figure 4C). Notably, a recent study has identified that temozolomide and radiotherapy could induce DDIT4 and repressed mTORC1 activity in some glioblastoma cell lines (44). Thus, overexpression of DDIT4 by demeclocycline in BTIC could be beneficial for glioblastoma patients. Interestingly, when we interrogated glioblastoma databases we found that elevated level of DDIT4 expression was associated with the longevity of glioma patients (Figure 5).

### Comparisons of Demeclocycline With Other Tetracyclines on BTIC Growth *in vitro*

**Figure 6:**
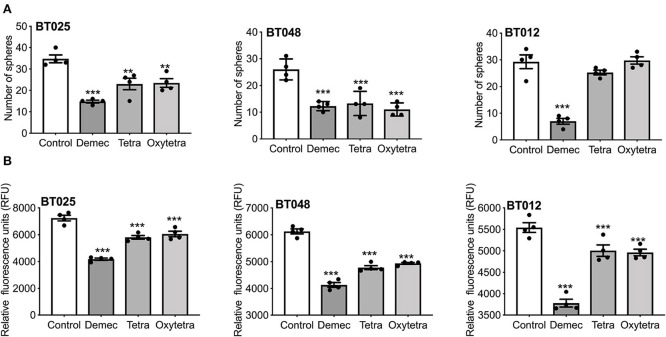
Effects of various tetracyclines on BTIC growth in culture. **(A)** Three glioblastoma patient-derived BTIC lines were exposed to 10 μM each of demeclocycline (demec), tetracycline (tetra) or oxytetracycline (oxytetra). **(B)** AlamarBlue® assay shows effect of demeclocycline, tetracycline and oxytetracycline on different BTIC lines with divergent genetic mutations, corroborating the reduced sphere-forming capacity of BTICs to varying extents across different lines. **p < 0.01, ***p < 0.001 compared to control (1-way ANOVA with Tukey's multiple comparisons). Error bars represent s.e.m. (*n* of 4).

We compared two other tetracyclines (tetracycline and oxytetracycline) to demeclocycline. We subjected three BTIC lines generated from glioblastoma patients with divergent genetic mutations, using demeclocycline as a positive control. Growth was assessed at 72 h after plating 10,000 cells/well in 96-well plate, using 10 μM of each drug. Figure 6A shows that while tetracycline and oxytetracycline reduced the sphere-forming capacity of BTICs to varying extents across different lines, demeclocycline inhibited the sphere-forming capacity of BTICs consistently across all lines. These results were corroborated by alamarBlue® assays (Figure 6B) and cell counts (data not shown).

## Discussion

Tumorigenesis not only alters the surrounding microenvironment, but is regulated by it (45, 46). Unfortunately, immune cells in the high grade glioblastoma (GBM) microenvironment generally assume tumor-promoting roles (47–51). Under the influence of GBM, microglia/macrophages are immunosuppressed and may even contribute to GBM invasion (52–54). BTICs are thought to help enforce immunosuppression (11, 12, 55–58). Thus, we have sought to sway the microglia/macrophage interaction with BTICs toward an anti-tumor phenotype. Via a drug screen of currently available medications, we discovered that amphotericin B could activate blood-derived monocytes to suppress BTIC proliferation and induce differentiation (26). However, amphotericin B is unlikely to be used as an immunostimulator for intracranial disease because of its substantial toxicity at high doses (59). Our attention turned to another drug found on the screen, meclocycline, and its more clinically attractive derivative, demeclocycline.

Demeclocycline is clinically attractive for several reasons. It has already been used for the treatment of bacterial infections and as a treatment for the syndrome of inappropriate antidiuretic hormone (SIADH) in humans (60, 61). A recent study showed that demeclocycline was also a promising contrast agent for the intraoperative detection of brain tumors (62). Moreover, when we exposed neural cells to demeclocycline, no significant toxicity was noted. Hence, its application as a drug to treat intracranial disease such as glioblastoma is more conceivable than with amphotericin B or meclocycline.

We first characterized the *in vitro* ability of demeclocycline to activate monocytes as these cells in the circulation could enter a glioma tumor to become macrophages (63). Thus, exposure of monocytes to an immunostimulator could theoretically result from systemic administration of a drug such as demeclocycline. Alone, demeclocycline did not increase TNF-α secretion by monocytes, a measure of monocyte activity. However, in the presence of primers such as IL-1β and IFN-γ, cytokines that are commonly elevated in glioma patients (38, 64), demeclocycline had stimulatory properties beyond that of when either IL1β or IFN-γ were administered alone. As confirmation of the immune-stimulatory effect of demeclocycline in the presence of a priming condition, similar results were seen when a conventional stimulator, LPS, was added. Also, the promotion of migration in demeclocyclineexposed monocytes supported the notion that this drug was an activator. To verify these results, the experiments were recapitulated with mouse macrophages. As another measure of the immunestimulatory capacity of demeclocycline, it was shown that conditioned medium collected from monocytes exposed to demeclocycline even in the absence of stimulators could decrease BTIC sphere formation. Given that temozolomide is the frontline chemotherapy for glioblastoma, this adds to the promise of demeclocycline as an additional treatment modality in glioblastoma.

Importantly, demeclocycline is not only an immune-stimulator, but can independently decrease BTIC viability, as indicated by alamarBlue and sphere formation assays. As with present treatments, subgroups of glioblastoma patients will be more sensitive to certain treatments based on factors such as genetics and previous treatments (65, 66), which may be true of the cell lines derived from those tumors.

To elucidate the mechanisms behind BTIC inhibition by demeclocycline, we employed a microarray analysis. This analysis has implicated several genes known to be involved in glioma or cancer progression (i.e., proliferation, invasion, metastasis) such as TGFB1II (39), FZD5 (40), EMR2 (41), ROMO1 (42), and BCL3 (43) which were significantly down-regulated with demeclocycline treatment in three BTIC lines (Figure 4). On the contrary, genes that negatively regulate Notch signaling, ATK signaling or mTOR activity, such as CHAC1, DDIT4, CLEC2A, were up regulated with demeclocycline treatment across all three BTIC lines (Figure 4). Taken together, these results support the use of demeclocycline as an anti-GBM treatment alone, or as an immunostimulatory agent acting on monocytes and macrophages, and potentially microglia if demeclocycline gains entry into the CNS.

In summary, we have identified a novel, potentially clinically compatible stimulator of monocytes that also has direct inhibitory actions on BTICs. This study has served as the basis for future work in which we will determine the safety and efficacy of demeclocycline in preclinical investigations, with the hopes to expand its use into humans.

## Data Availability Statement

The datasets generated for this study can be found in the GEO accession number GSE81515.

## Ethics Statement

All experiments with human cells or resected brain specimens were conducted with approval from the Conjoint Health Research Ethics Board, University of Calgary, with written informed consent from the human subjects.

## Author Contributions

SS, YL, and RM provided data for the response of BTICs to demeclocycline, other tetracyclines, and monocyte conditioned media. KR, CP, and JW provided data of the response of myeloid cells to demeclocycline. MK and PB conducted the bioinformatics and interrogation of databases. SS and VY oversaw the entire project and completed the editing of the manuscript after input from all co-authors.

### Conflict of Interest

The authors declare that the research was conducted in the absence of any commercial or financial relationships that could be construed as a potential conflict of interest.

## Supplementary Material

Table S1 Dysregulated genes after treatment BTICs with demeclocycline.

Figure S1 Clustering of dysregulated genes in BTICs after treatment with demeclocycline. Clusters of up-regulated **(A)** or down-regulated **(B)** genes were generated by the PANTHER classification system based on their functions. Up-regulated **(C)** or down-regulated **(D)** genes in corresponding categories are listed in the tables. Mean fold change (FC) is calculated for the three BTIC lines following treatment with demeclocycline versus no treatment.
