# Supplementary figures and images for: Demeclocycline Reduces the Growth of Human Brain Tumor-Initiating Cells: Direct Activity and Through Monocytes
Source: Front Immunol. 2020 Feb 21;11:272. doi: 10.3389/fimmu.2020.00272 (PMC7047330; doi:10.3389/fimmu.2020.00272)

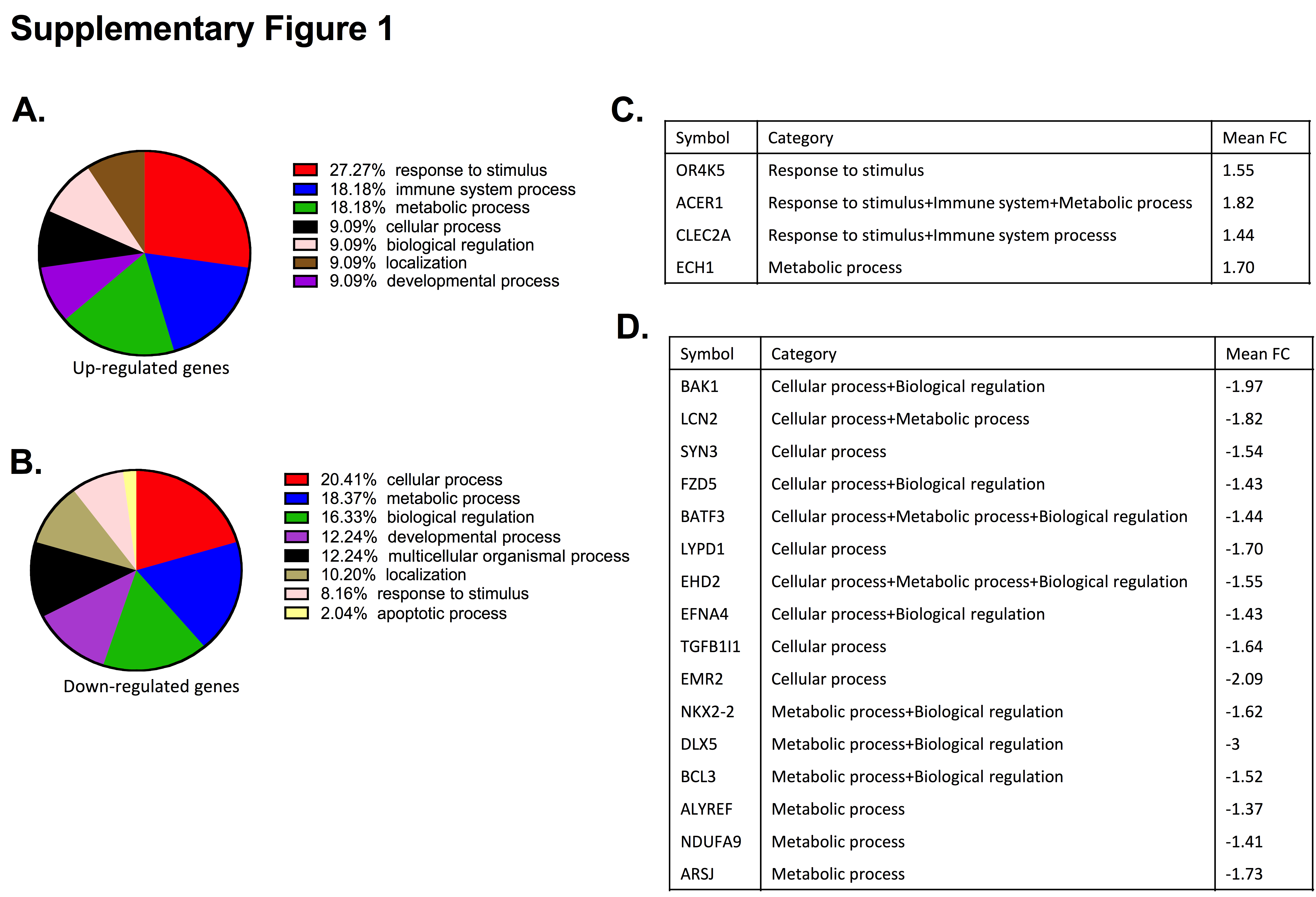

Supplement: Figure S1 — Clustering of dysregulated genes in BTICs after treatment with demeclocycline. Clusters of up-regulated (A) or down-regulated (B) genes were generated by the PANTHER classification system based on their functions. Up-regulated (C) or down-regulated (D) genes in corresponding categories are listed in the tables. Mean fold change (FC) is calculated for the three BTIC lines following treatment with demeclocycline versus no treatment. [file Image_1.TIFF]
